# The Possible Role of Transplacentally-Acquired Antibodies to Infectious Agents, With Molecular Mimicry to Nervous System Sialic Acid Epitopes, as Causes of Neuromental Disorders: Prevention and Vaccine Implications

**DOI:** 10.1080/17402520600801745

**Published:** 2006

**Authors:** André J. Nahmias, Susanne Beckman Nahmias, Dan Danielsson

**Affiliations:** 1 Pediatric Infectious Diseases Epidemiology and Immunology Division Department of Pediatrics and School of Public Health (emeritus) Emory University Atlanta GA 30322 USA; 2 Georgia Parents for Responsible Health Education PO Box 15006 Atlanta 30333 USA; 3 Department of Clinical Microbiology Örebro University Hospital Örebro S-701 85 Sweden

## Abstract

Proof of causality of most neuromental disorders (NMD's) is largely unavailable. Lessons from four-decade investigations of the epidemiology, immunology, pathogenesis, prevention and therapy of perinatal infectious agents, which invade directly the nervous system, have led us to propose a new indirect effect hypothesis: maternal transplacentally-acquired antibodies, to agents with epitope molecular mimicry with the developing nervous system, can cross the fetus/infant's blood–nervous system barriers to cause NMD's, clinically manifest years later.

Further rationale is provided by relevant evolutionary/developmental (EVO–DEVO) considerations—applicable also to some vaccines. The hypothesis is being tested in: (a) older pregnancy studies with available maternal and newborn sera, and follow-up of the progeny for NMD's; and (b) NMD registry individuals linked to their stored newborn blood spots. Preliminary results support a possible role for schizophrenia of high-tittered antibodies to some agents (toxoplasma, influenza and herpes simplex type 2 virus).

A model that includes likely genetic and postnatal influences is schematized and a list of putative agents and factors, based on varying rationales, is tabulated. In case pilot studies are confirmed, the identified agent(s) and antibodies would need to be tested in new prospectively enrolled pregnant women, so as to establish further risk factors leading to possible preventive modalities.


*Clinical & Developmental Immunology*, June–December 2006; 13(2–4): 167–183

Taylor & Francis

Taylor & Francis Group

### The possible role of transplacentally-acquired antibodies to infectious agents, with molecular mimicry to nervous system sialic acid epitopes, as causes of neuromental disorders: Prevention and vaccine implications

ANDRÉ J NAHMIAS[1], SUSANNE BECKMAN NAHMIAS[2], & DAN DANIELSSON[3]

[1]*Pediatric Infectious Diseases, Epidemiology and Immunology Division, Department of Pediatrics and School of Public Health (emeritus), Emory University, Atlanta, GA 30322, USA,* [2]*Georgia Parents for Responsible Health Education, PO Box 15006, Atlanta 30333, USA, and* [3]*Department of Clinical Microbiology, Örebro University Hospital, S-701 85 Örebro, Sweden*

**Abstract**

Proof of causality of most neuromental disorders (NMD's) is largely unavailable. Lessons from four-decade investigations of the epidemiology, immunology, pathogenesis, prevention and therapy of perinatal infectious agents, which invade directly the nervous system, have led us to propose a new indirect effect hypothesis: maternal transplacentally-acquired antibodies, to agents with epitope molecular mimicry with the developing nervous system, can cross the fetus/infant's blood–nervous system barriers to cause NMD's, clinically manifest years later.

Further rationale is provided by relevant evolutionary/developmental (EVO–DEVO) considerations—applicable also to some vaccines. The hypothesis is being tested in: (a) older pregnancy studies with available maternal and newborn sera, and follow-up of the progeny for NMD's; and (b) NMD registry individuals linked to their stored newborn blood spots. Preliminary results support a possible role for schizophrenia of high-tittered antibodies to some agents (toxoplasma, influenza and herpes simplex type 2 virus).

A model that includes likely genetic and postnatal influences is schematized and a list of putative agents and factors, based on varying rationales, is tabulated. In case pilot studies are confirmed, the identified agent(s) and antibodies would need to be tested in new prospectively enrolled pregnant women, so as to establish further risk factors leading to possible preventive modalities.

**Keywords:** *Infection, neuromental disorders, transplacental antibodies, molecular mimicry, vaccines*

"Tracing the causes of mental illness is a much more difficult task than locating structural damage to the brain" Eric R Kandel "In Search of Memory" (2006)

### Introduction

We were inspired to emulate the more personal approach of Kandel's personal journey of 50 years, rather than with the more stereotyped approach to writing scientific papers. Of particular relevance is how Kandel's initial interest in psychiatry led him, after developing the neuroscience of memory in lower biological forms, to return to issues about neuromental processes in people.

Similarly, our early experience in clinical medicine, epidemiology and immunology, with a focus particularly on perinatal infections, has taken us—over the past four decades—on a circuitous journey, to take on new challenges related to the causality, diagnosis and prevention of some neuromental disorders (NMD's). During our journey, we have also explored various aspects of evolution (viruses, immunology and sexually transmitted infections) and of development (placenta and immune systems). Now that evolution

Correspondence: A. J. Nahmias, 747 Houston Mill Rd Apt 3, Atlanta, GA 30329, USA. Tel: 1 04 636 8984. Fax: 1 404 325 0880. E-mail: nahmias@bellsouth.net

ISSN 1740-2522 print/ISSN 1740-2530 online © 2006 Taylor & Francis
DOI: 10.1080/17402520600801745

and development have been linked into the new discipline of EVO–DEVO, we convey much of the information and rationale relevant to our hypothesis within the broader EVO–DEVO perspective of Biology.

We had been familiar with the NMD's associated with many infectious agents acquired *in utero*, intrapartum and during infancy, which—following HIV/AIDS parlance, we will also consider altogether as perinatal infections. Occasionally we would also be asked to consult on psychiatric diseases presenting as infectious diseases (Wurtz 1998). As we became more acquainted with the many newer advances in neuroscience, molecular genetics and psychiatry (Charney and Nestler 2004), we were struck by the scarcity of proven causality, specific laboratory or radiological diagnostic tests and preventive approaches for a large number of NMD's. As medical scientists also involved in the epidemiology of global infectious diseases (Nahmias et al. 1990) we had not been as aware of the enormity of the total burden of NMD's in the world. The World Health Report (2001) estimated that NMD's represented at least 12% of all diseases, with around 10% occurring by the age of 12 years, and at least twice that many in adolescents and adults. Quality-of-life and Disability Lost Years (DALYs) measurements demonstrate the enormous impact on afflicted individuals. Olness (2003), from a more clinical perspective, has also confirmed that the effects on neural development leading to cognitive impairment represents a worldwide epidemic. Assuming that there is a no great variability among different world populations (in case of schizophrenia the rate globally is around 1%), since few causalities are known or preventive measures available, one can estimate that over 10–20 million of the > 100 million babies born this year are destined to develop one or more NMD's in their life. These numbers are higher than the estimates of the global impact of the combined number of children born every year who will develop tuberculosis, malaria and HIV, or of neonatal infections alone (Stoll 1997).

It is not hard to predict that the large amount of basic neuroscience knowledge unraveled in 1990s "Decade of the Brain" and since then (Nature eds. 2002; Kandel 2006), will result in many more novel treatments for afflicted individuals, than in the development and application of new preventive modalities. We base this prediction on our experience of over 20 years with HIV/AIDS (Schinazi and Nahmias 1988), which provides a good infectious model of a chronic disease, with similar attached stigmas as several NMD's. More than 20 antiretrovirals are now available for treatment of the viral infection, whose most effective impact in pediatrics has been in the primary type prevention of maternal-child transmission. However, despite generally good effectiveness for the treatment of children and adults, there is no cure yet of the affected individuals, as is the case for many therapies for NMD's. Both disease entities have common problems as to compliance, cost and side effects, often associated with drug interactions. There are also other similarities for both entities: (a) drugs for treatment have generally been poorly studied in children and pregnant women, in whom possible ill-effects on the progeny are of concern; (b) there is a continuous need for an infrastructure of health personnel, education, and counseling; and (c) for both entities, even if it were possible to treat every clinically affected individual, unless successful prevention modalities are found and applied effectively, more cases needing therapies will accrue to those already on chronic treatments. One possible difference is that, while there has been a great deal of HIV/AIDS "activism"—helping to provide the funds and the drugs for treating individuals in developing countries (unfortunately as yet, only for a small proportion), there is no apparent comparable activism for drugs to treat NMD's.

Our hypothesis, whose specific aim is to ascertain possible infectious agents that can be primarily prevented, is first provided as a general guide for the reader in Figure 1. This simplified figure is made more comprehensive in two later figures, comprising (a) a list of putative infectious agents and factors to study, based on preliminary results and (b) a model of the possible multifactorial mechanistics operating in space and time—in the nervous system and during, as well as after, the perinatal period. Thus, rather than a direct pathogenetic effect of infectious agents on the nervous system of the fetus/infant, the hypothesis proposes an indirect effect. In this case, it is the antibodies of the mother to infectious agents, with molecular mimicry to the developing nervous system, which are transferred transplacentally, crossing the blood–nervous system barriers more permissively to cause a proportion of some NMD's of the fetus/infant that are most often not clinically manifest until later years. We next present how this hypothesis was developed, as a longtime search for "O" infectious agents. This more personal model, (primarily by AJN,) will be followed by EVO–DEVO considerations. Relevant information is also used to identify putative causal infectious agents, vaccines, and other pathogenetic factors. The clinico-epidemiological and laboratory methods that can be used to test the hypothesis, first used by US and Danish colleagues, are then presented with our own interpretation of the related preliminary data. Finally, possible follow-up studies of any confirmed pilot studies are suggested that would help to evaluate approaches for establishing other risk factors and possible preventive modalities, as suggested from the success with the many perinatal infections presented below.

### Our longtime search for the "O" infectious agents associated with neuromental disorders

We were first introduced as long as four decades ago to the NMDs associated with several infectious agents

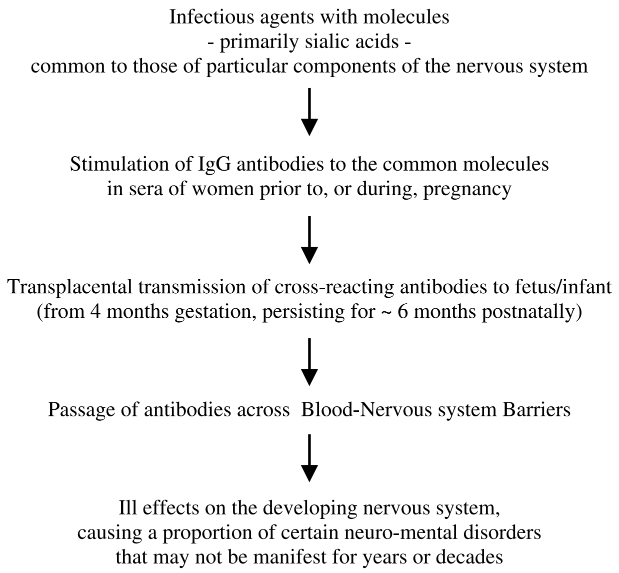

Figure 1. A simplified version of the Gestational Neuro-Immunopathology (GENIP) Hypothesis, emphasizing that the NMD that might occur (have its origin?) during neural development of the fetus/infant may not become clinically manifest until years or decades later.

acquired intrauterine, intrapartum or postnatally during infancy. Most of the bacterial causes of many of these infections had been generally diagnosable, e.g. neonatal bacterial meningitis, and some antibacterial therapies had become available, preventing some—but by no means all—of the fatalities or neuromental sequelae demonstrable later in life. The best success achieved for over half a century relates to congenital syphilis, whereby penicillin treatment of the mother diagnosed before, or during, pregnancy prevented fetuses and newborns from experiencing acute disease manifestations, as well as the later sequelae detectable sometimes decades later, e.g. neurosyphilis (Nahmias et al. 1994a).

With the ability to grow viruses in culture, by the 1950s, and to diagnose some parasitic diseases in research laboratories, neonatal sequelae were being recognized in individuals, infected primarily *in utero*, by Toxoplasma, Cytomegalovirus and Rubella virus. When, around the mid-1960s, one of us co-discovered a new Herpes simplex virus (type 2), most frequently transmitted intrapartum (Nahmias et al. 1970a), it became apparent that its clinical manifestations, including congenital malformations and/or neuromental sequelae, often mimicked those of the other three organisms and vice versa (Nahmias et al. 1970b; Nahmias and Visintine 1979). To obtain more data of medical and public health importance, an alliance with workers—at the time termed Communicable Disease Center (CDC)—was formed to study all four agents together, whenever one of the four was suspected clinically. We eventually grouped these four perinatal infectious agents into the acronym TORCH (Nahmias et al. 1971), which has been widely used since (Nahmias and Keyserling 1984). The "O" was inserted presciently to convey the message that there would likely be other perinatal agents with overlapping symptomatologies, including NMD's, yet to be discovered. We put much effort, including attempts to detect new viruses by electron microscopy (Lee et al. 1983), but failed ourselves to find any "O" agents. Other new agents, which are not readily diagnosable as are bacteria, were indeed discovered by various workers, including parvoviruses, hepatitis viruses and HIV. However, of these, only perinatal HIV fulfills the requirement of an infection that can cause NMD's (Schinazi and Nahmias eds. 1988). Based primarily on observations in animals or older-than-infant humans, some investigators have implicated—without finite proof— the role of Borna virus (Iwahashi et al. 1997), human endogenous retroviruses HERV (Karlsson et al. 2001), and other infections (Shoenfeld and Rose eds. 2004), as direct agents or via autoimmune mechanisms in the etiology of some NMD's. In case of HIV perinatal infection, we now appreciate, not only the severe early-age encephalitis, but also the later neuromental dysfunctions in HIV/AIDS children and adolescents, even those receiving successful antiviral therapy (Castellon et al. 2006). It should be emphasized that the pathogenesis of neural involvement, using the more recent molecular and cellular methods, are best known for HIV, than for any of the TRCH agents.

The medical and public health focus on the TRCH agents possibly helped in the improved development of specific, often rapid, diagnosis (Remington et al. 2006). Most important has been the advent of successful approaches for the prevention of syphilis, rubella, herpes simplex, HIV and toxoplasma—best accomplished with the first two types of preventive modalities. These experiences provide models that could be applied for the prevention of NMD's whatever the cause. *Primary prevention* is the most

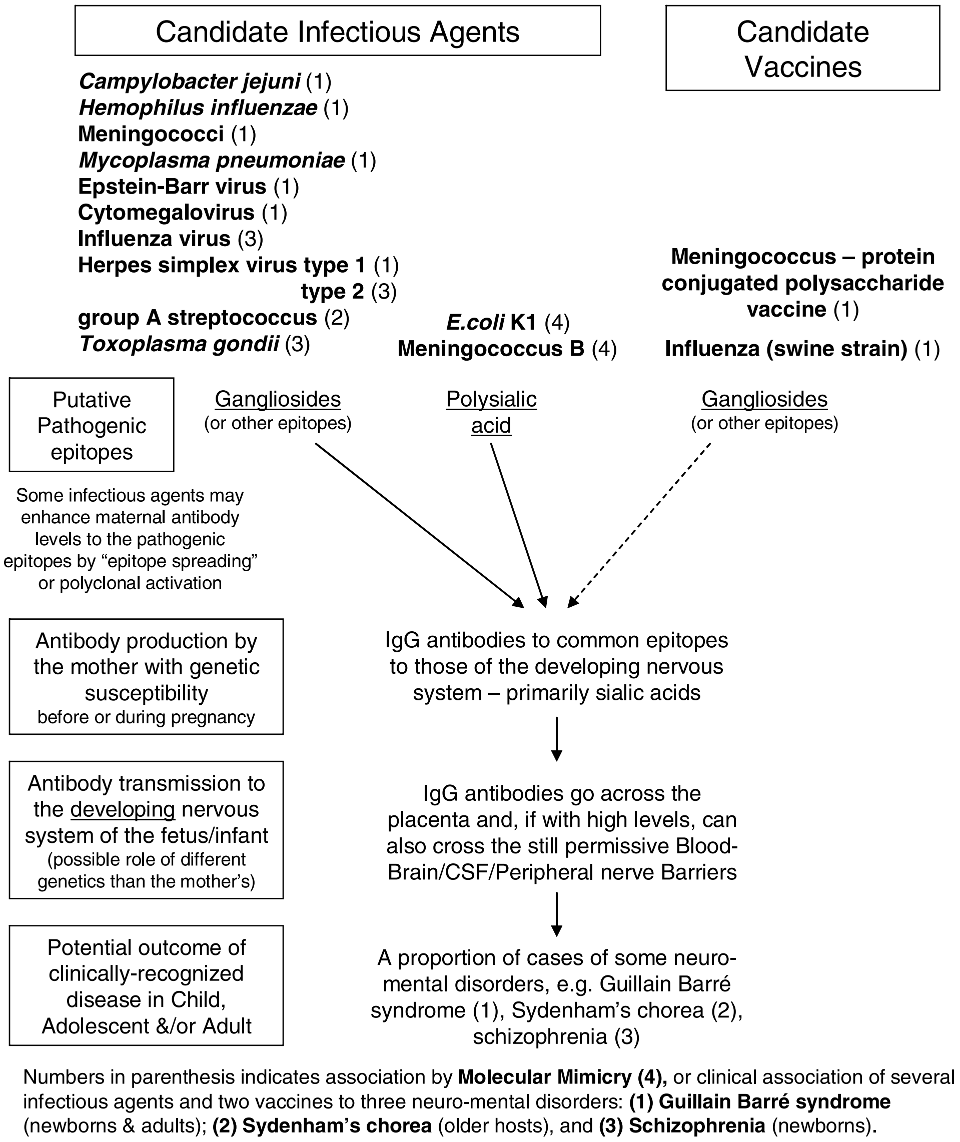

Figure 2. A more detailed version of the GENIP Hypothesis, listing candidate infectious agents and vaccines, and pathways from mother to fetus/infant, leading to NMD in later life. The rationale for selection of the candidate factors, detailed in the text, is based on molecular mimicry, (and/or) their association with various NMD in newborns and older hosts.

desirable, with prevention being accomplished, either with general childhood vaccination (rubella), or by diagnosing the maternal infection, treating the mother, and/or performing a cesarean section (syphilis, toxoplasma HSV, HIV and group B streptococcus). *Secondary prevention* is accomplished by preventing an established infection to go on to manifest diseases, by effective treatment of the asymptomatically infected infants, e.g. several bacterial infections. *Tertiary prevention* attempts to stop the clinical manifestations from progressing to more severe disease or death (many of the above agents)[1]. Primary or secondary prevention of genetic causes, in our estimation, would be even more of a problem than for a direct or indirect infectious etiology, particularly as regards ethical and socioeconomic considerations.

The specific diagnosis of the large majority of NMD's, except for those with single gene mutations (e.g. fragile X syndrome, or a minority of cases of the autism spectrum), is usually by combining symptoms and/or sign as listed in DSM-IV-TR (2000). Infectious disease specialists are more used to diseases, which have known causes and specific diagnostic methods, although having been plagued at times by initial misinterpretation of the true causal agent, e.g. *Hemophilus influenzae*, for decades supposed to be the cause of influenza disease due to a virus. Frustrating to many of us, currently, is the inability to find a likely infectious cause of a severe immunopathological disease (Kawasaki) that may lead to coronary artery complications, for which i.v. gammaglobulin serves as a tertiary type of prevention.

Figure 3. A model depicting the WINDOW for high or low levels of transplacentally-acquired IgG/antibodies to cause NMDs in relation to: (1) development over time of neurological components and/or functions; (2) change over time in the permissiveness of Blood–Brain–CSF-Barriers; and (3) a potential threshold for the development of dysfunction with time, dependent on genetic, epigenetic and postnatal environmental influences.

It is well appreciated that for many infectious, autoimmune, neuromental and other types of disorders, a genetic predisposition is likely to be an important factor. Although genetics or environmental factors alone can cause a proportion of different NMD's, genetic and postnatal environmental influences are likely to be contributory (Faraone et al. 1999), as noted later in our model. Most often, postnatal environmental, including socioeconomic, factors are considered, without inclusion of the gestational[2] environment, during which a major portion of neural development occurs. The neurodevelopmental hypothesis had been earlier postulated by Weinberger (1996) and Brown (1999). However, our GENIP hypothesis also points to the importance of researching, not only neural system genetics, but also immuno-genetics, e.g. the genetically influenced responsiveness of certain pre-pregnant or pregnant women to produce high titers of IgG antibodies to polysaccharide (PS) antigens.

A century of postmortem studies of the brain of mentally ill persons has failed to reveal the clear, localized lesions observed in such chronic diseases as Huntington's chorea, Parkinson's disease or amyotrophic lateral sclerosis (Kandel 2006). Even in the advent of newer means to detect anatomical and functional abnormalities of the brain, e.g. functional MRI, those methods have not yet allowed specific diagnosis to be made. It is most often difficult to differentiate effects from causes. In contrast, studies of the cerebrospinal fluid (CSF), brain or other neural tissues usually demonstrate direct or indirect markers of the great majority of infectious agents. The lack of pathological markers is one of the main reasons why we have sought possible indirect effects of infectious agents, which may result from maternally-transmitted transplacental antibodies. We have learned over the years (Remington and Klein 2006) that, in case of perinatal infections, readily discernable major brain involvement (e.g. microcephaly with rubella and cytomegalovirus; hydrocephalus with toxoplasmosis), occur primarily during the first 3–4 months of gestation—a period also of great neurogenesis activity. After 4 months gestation, despite the fact that a TRC agent was still transmissible across the placenta until delivery (sometimes even more readily transmitted as with toxoplasma), the clinical effects are usually less common or severe, with ocular or NMD's clinically manifest months or years after birth. We connected these observations with the fact that, around 4 months gestation is also the time when maternal antibodies begin to be transmitted transplacentally.

There have been numerous reviews (Shoenfeld and Rose 2004; Molina and Shoenfeld 2005)—as well as the reports at the Lausanne Conference on the subject in 2005, which have denoted a large number of associations that have been made between infectious agents or vaccines, and autoimmune diseases and/or NMD's. Despite considerable efforts, causality has been extremely hard to prove. In case of acute infections as suspect initiators, the best links are made when the infection is clinically manifest, or when a vaccine is administrated, and a NMD (or other ill-effect) is observed within a month or at most two. Side-effects of the new protein-conjugate meningococcus PS vaccine, for instance, were mostly identified prospectively for less than two months (MMWR 2006). In either case—infection or vaccine—the ill-effects may be due to other coincidental causal stimuli of the disease; this necessitates knowledge of the regular incidence of the implicated disease entity in similar populations and locations, as used for instance by the Vaccine Safety Data Link (Chen et al. 1997). If the infection or vaccine ill-effects are demonstrable years later, or were due to epitope spreading or polyclonal activation, the real culprit will most likely be missed. Similarly, an association made on epidemiological grounds without laboratory confirmation may not detect the true causal factor, e.g. the association made of a higher risk of schizophrenia in babies born during the influenza season (Brown 1999), (see also results of Brown et al. (2004) presented in Testability section.)

The two infectious agents most widely accepted as causes of NMD's happen to have first been detected within a period of a month, or so, after symptomatic disease: (1) group A *Streptococcus pyogenes* infection, e.g. tonsillitis or scarlet fever, with secondary Sydenham's chorea and rheumatic heart disease; (2) *Campylobacter jejuni* diarrhea, with the later manifested Guillain Barré syndrome. Both entities have been associated with molecular mimicry of sialic acids common to those of the nervous system (Hughes et al. 1999; Kirvan et al. 2006). Together with the EVO–DEVO considerations noted later, these data have provided an important stimulus to focus on sialic acids as a likely major "pathogenic epitopes", for our GENIP hypothesis (Figure 2).

Behavior is likely to be linked to our genes, as well as environmental influences, including maternal alcohol and drug abuse during pregnancy (Faraone et al. 1999; Drayna 2006) A genetic, particularly polygenetic, or epigenetic, causal association with any NMD represents a major effort, despite the availability of a large number of new molecular genetic tools. This has been exemplified recently in case of neuregulin 1 (NRG1)—a leading schizophrenia susceptibility gene found by most, but not all investigators, to be associated with this NMD (Harrison and Law 2006). These authors note that unequivocal proof of NRG1 as a schizophrenia gene remains a daunting task, as several variants appear to be non-coding, regulatory genes, which are likely to be triggered by epigenetic or environmental factors. An upset in the regulation of the gene could also cause increased generation of a protein involved in neuropathology (Law et al. 2006). The presence of NRG1 in several organs, and its involvement in a variety of developmental and other functions of the nervous system (neuronal migration, synaptic formation and glial differentiation among others) add to problems of arriving at proof of causality.

Other considerations learned from our infectious disease experience are: (1) a single agent may cause different disease manifestations, e.g. pneumococcus can cause otitis, sinusitis, meningitis, etc. Similarly, the co-morbidity of more than one NMD in a single individual's life, whether at the same or different times is not an uncommon clinical experience, and (2) the disease may be caused by many infectious or non-infectious agents, e.g. pneumonitis can be due to a large number of different viral, bacterial, fungal or parasitic infectious agents. It therefore would be unlikely that any of the candidate infectious agents whose rational for testing is described later, would cause 100% of the NMD being investigated. Nevertheless, it would be most worthwhile to develop some preventive measure for even a small proportion of any severe, debilitating NMD, as was successfully accomplished for the NMD sequelae—including schizophrenia cases—associated with prenatal rubella virus (Brown et al. 2001).

An EVO–DEVO overview is particularly helpful to bring together a variety of multifactorial aspects, including relevant development and phylogenetic aspects of the placenta and of the immune and nervous systems. EVO–DEVO is also important as related to the applicability of certain animal models to particular aspects of the human condition, in view of the differences in the placental anatomy and physiology. The mental processes involving the brain cortex are markedly different among lower species. Kandel (2006), points out that studies in lower animals related to cognitive functions are not as likely to prove as helpful, as those related to emotional disorders, such as anxiety problems or depression, or to memory disorders related to the hippocampus. This is attributed to cognition being associated with the human cortex that developed over a recent brief period of evolution, whereas emotion and memory are associated with brain structures that have not substantially changed, over long evolutionary periods, between lower and higher species. Nevertheless, as noted later, pathogenetic studies on the mouse cortex have provided important means to relate certain genes to the evolution of the human cortex (Hill and Walsh 2005; Walsh unpublished results 2006)

### Evolutionary/developmental (EVO–DEVO) perspective on relevant components of the GENIP hypothesis

For those of us interested in the phylogeny and development of the immune, nervous and reproductive systems, as well as of infectious agents, the linkage of the two (EVO–DEVO) has proved both intellectually rewarding and challenging. This new discipline has been reviewed in a book by Carroll (2005), who focused mainly on one of the major links between evolution and development—the *HOX* genes, which evolved over 640 million years ago, and have been found to possess gene homologies between drosophila flies and higher species, including humans. At the developmental level, *HOX* genes carry information about how embryonic cells are arranged in space and time, so that body cavities become organized, tissues differentiated and organs formed, all in their proper place and in proper sequences. The relative paucity of the expected number of genes in humans, as compared to the lower species—characterized in the Human Genome Project—has led to the concept that gene function is regulated by "switches", as noted decades earlier regarding the impact of the environmental milieu on *Escherichia coli* mutants. Epigenetic, as well as environmental factors, which can occur during gestation or postnatally thus renders causality much more complex to decipher than single gene mutations—already quite a complex task.

EVO–DEVO aspects of the reproductive system, including the placenta, remained obscure until *HOX* genes were found to be involved (Lynch and Wagner 2006). Thus, *HOX* gene homologies have been characterized in several of the reproductive organs in amphibians, birds, monotremes, marsupials and placental mammals. Novel *HOX* gene adaptations over the past 3 million years have been involved in fetal–placental–maternal immune interactions permitting the fetus not to be rejected or to cause harm to the mother. Such maternal–placental–fetus–infant immune interactions we called the "Great Balancing Act" (Nahmias and Kourtis 1997), as well as factors related to why pregnant women do not reject their fetus (Nahmias et al. 1998a), can be better explained now in the context of the *HOX* gene discoveries. Similarly, the embryology of congenital anomalies of body patterning is now being better understood (Goodman 2003).

Well appreciated, from the review in the book by Benirschke et al. (2006) is the existence of a large variety of anatomical and physical differences in the placenta of different mammalian species—lending again caution to which animal models to use. Of particular relevance to our antibody focus, are the marked differences in the selective transfer of antibodies and their immunoglobulin (Ig) classes in different species (Butler 2006). Thus, in humans, transplacental transfer only occurs with the IgG class and four subclasses ($IgG_{1,2,3,4}$); the colostrum (and breast milk), contains primarily IgA. In marked contrast, IgG does not cross the placenta of horses, pigs and cows, and is only found in the colostrum. That the IgM class, in which many "natural" PS antibodies to self are located, can be produced by the fetus/infant—yet is unable to go across either the placenta or the blood–nervous system barriers—suggests that this immune system was adapted to minimize the possible ill-effects of such IgM antibodies on the nervous system.

In humans, relevant EVO–DEVO features are the differences in the placental transmission of the four IgG subclasses (Malek et al. 1996), and of the antibodies to proteins or to PS (Nahmias and Kourtis 1997). Thus, antibodies to proteins, e.g. tetanus toxoid are of almost equivalent titers in the newborn at delivery to the mother's. On the other hand, $IgG_2$ and antibodies to PS antibodies, e.g. to pneumococcus PS (located primarily in $IgG_2$), are about two-third of the mother's levels in the newborn at birth. A corollary is that antibodies to proteins, being usually of higher titers, will persist in the infant's blood for up to 12–15 months, whereas the more commonly lower-tittered antibodies to PS last about 6 months in the infant.

A well-known model for our GENIP hypothesis is that of blood group incompatibilities (Fagiolo and Toriani-Terenzi 2003), which can be better comprehended from an immunological EVO–DEVO viewpoint: (1) Although less common population-wise, Rh incompatibility is a much more serious perinatal disease than ABO incompatibility, which tends to cause most frequently no, or mild, disease—although severe hemolysis effects can occur. As noted above, this is due in part to the more efficient transplacental transfer of antibodies to proteins (Rh is a protein on the red blood cells), than PS antibodies (A and B have PS on the RBCs). Furthermore, antibodies to proteins are boostable, so that the sensitized Rh-mother, if receiving RBC's of a later Rh + fetus, will develop very high IgG anti-Rh antibodies. In case of ABO incompatibility, the PS IgG antibodies will tend to be low or absent, as the IgG antibodies to PS antigens are not boostable. (2) The ABO blood group incompatibility is an excellent model for molecular mimicry (detailed later). The A + fetus will not produce anti-A antibodies (due to tolerance), but acquires anti-B antibodies—initially in IgM by one year of age and IgG by 2, or more years. This is due to the molecular mimicry of the AB PS to those of foreign *E. coli* bacteria, with similar types of mimicry occurring with other blood group antigens, e.g. the Lewis factor and some bacteria. Of possible related interest is that Insel et al. (2005) have reported that maternal fetal Rh and ABO blood incompatibility was a risk factor for schizophrenia, but only in male offspring.

Developmental aspects of the placenta, related to monozygotic (MZ) and dizygotic (DZ) twins, impact on the genetic interpretations of discordant results found between MZ and DZ twins for studies on NMD's, as well as other entities. This is due to several factors, including placental differences: the placenta of MZ twins is most usually monochorionic, and that of the DZ twins dichorionic (Benirschke et al. 2006). Placental blood vessel anastomoses often occur in the monochorionic placenta between twin A and twin B of a MZ pair, causing, the well-known feto–fetal transfusion syndrome or severe anemia in one of the twins. However, it is not fully appreciated that other proteins besides hemoglobin, that may affect one of the two identical twins, have been found by Bryan (1977) to differ in the newborn blood of MZ twins, and not DZ twins. (We have recently initiated studies in NMD discordant MZ and DZ twins, to expand these unique old findings and to evaluate differences in various antibodies and other factors—see Testability section later).

Most studies on twins do not record placental anatomy and pathology, as well as other non-genetic factors, such as obstetric and newborn complications that are more common in identical twins (Benirschke et al. 2006). Seldom reported are also other such possibly important variables as birth weight, gestational age, maternal or newborn infections, and whether deliveries were vaginal or by cesarean section. For instance, the initial data, suggestive of the intrapartum route of maternal-child transmission of HIV, was the finding of a greater infection rate in the first twin than in the second twin (Palca 1991). Furthermore, our experience and that of others (usually single case reports) indicate that only one of the two MZ twins becomes infected with a TRCH agent, or with HIV (Menez-Bautista et al. 1986).

Results of twin studies and of family studies generally tend to favor genetics and heredity interpretations. For instance, in case of schizophrenia, the overall finding has been that, when a MZ twin has this serious NMD, the other identical twin has a 50% risk; whereas among DZ twins, the risk—when one is afflicted—is only 15% (Kandel et al. 2000). This is interpreted usually as indicating genetic influences, although placental and other factors noted above may be involved. In addition, if one looks at the other side of the coin, the finding that 50% of MZ twins are not afflicted by schizophrenia strongly support the likelihood of non-genetic influences; when this is noted, mainly postnatal environmental, including hormonal or socioeconomic factors are usually considered.

Studies performed in family members, particularly close relatives, who show a greater risk of a similar or other, NMDs are often interpreted as providing further evidence of genetics. However, familial is not necessarily synonymous with hereditary (Torrey and Yolken 2000). Long-term environmental factors such as toxins, e.g. lead, in the household could be involved. It is also well appreciated that the same chronic infection, or one recurring, in a woman during repeated pregnancies may affect more than one sibling. For example, we have found cases in which an HIV infected pregnant woman, who has experienced five consecutive pregnancies, may only infect two of the children with similar clinical manifestations; without knowledge of the viral causation of AIDS, genetic factors would likely be implicated. Nevertheless, as infectious disease specialists, we are convinced that genetic predispositions, whether related to innate or adaptive immunity, are most likely to play a role in defining the range of subclinical, to severe and fatal, disease with most infectious agents, e.g. pneumococcus and poliovirus. Current single-nucleotide polymorphism (SNP) studies should provide further evidence of the genetics, and the risk potential of particular infections, as is being currently performed for NMD's, cancer and other disease entities.

The immunology of human infections with relevance to what is now considered EVO–DEVO, was reviewed over 20 years ago in two books we edited (Nahmias and O'Reilly 1981,1982), as well as reports on the ontogeny of immunity to some infectious agents (Nahmias 1992; Nahmias et al. 1994b; 1998b). With the discovery of TOLL receptors, found from drosophila to humans, and the advances in molecular genetics, much knowledge has been obtained of the innate immune and the adaptive immune systems (Flajnik and Du Pasquier 2004; Kasahara et al. 2004) The interactions between the two types of immunity had to adapt to each other as the host species evolved—similar to the co-evolution of old families of some viruses to their evolving host species (Nahmias and Reanney 1977). Thus, as new immune cells and systems developed in evolving hosts, the new ones would have to interact with the older ones. One of our more recent studies, for instance, suggests that the protective immunity of PS antibodies developed early, before the development of the classical T cell-MHC1 and 2-dependent production of antibodies to proteins. Thus, we showed—at least in the mouse—that antibody production to pneumococcus PS antigens was CD1-dependent, and also required, as has also been reported by other workers (Snapper et al. 2001), a T cell which does not interact with characteristic MHC1 or 2 (Kobrynski et al. 2005)[3].

Of relevant evolutionary interest, particularly at the molecular level, is a whole issue of the "Developmental and Comparative Immunology" journal (Butler ed. 2006) on the antibody repertoire from fish to man. In that same issue Marchalonis et al. (2006) summarized antibody evolution as follows: "Overall, antibodies of jawed vertebrates show tremendous individual diversity, but are constructed in incorporating design factors that arose with the evolutionary emergence of the jawed vertebrates and have been conserved through at least 450 million years of evolutionary time". These conclusions would suggest that evolution had "all the time in the world" to develop immune mechanisms that would help to protect young children from severe, often fatal diseases. Nevertheless, children below 2 years of age cannot generally produce protective IgG antibodies to PS-encapsulated bacteria such as *H. influenzae* type B, pneumococci, meningococci, *E. coli* K1, group B streptococci and tuberculosis (disseminated disease).

Why then, are young children left vulnerable to these virulent, often fatal or debilitating bacteria? On the other hand, if infected *in utero*, at birth or soon thereafter, the fetus/infant can produce all classes of Ig, as well as antibodies to specific protein antigens they encounter. Using ELISPOT technology (Lee et al. 1991) we found that, even 6 months-gestation live newborns can produce high numbers of IgG-, IgM- and IgA-secreting cells and protein antibody-secreting cells to syphilis (Stoll et al. 1993). ELISPOT technology (Lee et al. 1989) measures the circulating B cell components of the infant's blood which are in the process of producing Ig and antibodies. Serum measurements are unreliable, as one cannot differentiate newborn production of IgG, from maternal transplacentally-acquired IgG; measurements of IgA in serum also give false information on the infant's mucosal IgA production, as secretory IgA is broken down in the liver (Nahmias et al. 1991).

From the concatenation of observations presented above, we conclude, barring further data, that mechanisms of both passive (transplacental acquisition) and active IgG antibodies to PS antigens by the fetus/young child—with no boostability—have been adapted throughout evolution to protect our species from the ill-effects of certain high-titered IgG antibodies to PS, most probably due to their molecular mimicry with those of the nervous system. This interpretation is important also because of possible correlates. One is that some caution should be given to protein-conjugated PS vaccines, used so successfully to decrease markedly *H. influenzae* type B, pneumococcal and meningococcus C disseminated diseases. Another advantage to these vaccines is that the protection conveyed has also been shown to occur not only in the vaccinated individuals, but also in non-vaccinated persons, by epidemiological mechanisms termed "herd immunity" (Musher 2006). We do not wish to see further decreases in the availability and research on vaccines, as reviewed by Klein and Helms (2006). Production of conjugated vaccines varies from that for the more traditional older vaccines—whether inactivated or attenuated that were developed to mimic, if not surpass, the immunity produced by natural infection. However, the protein-conjugated PS vaccines, and others planned for other bacteria represent relatively novel approaches to the production of vaccines, prepared to overcome what appears to be evolutionarily developed immune mechanisms. The concerns expressed with older vaccines, e.g. measles, or those containing mercury, in regards to autism, have not been validated. Even though a small number of cases of Guillain Barré syndrome have been recently associated with the new protein-conjugate vaccine with four subgroups of meningococci (A, C, Y and W135) (MMWR 2005, 2006), causality has not been proven conclusively. This recent experience, however, suggests that follow-ups of the newer vaccines be performed for longer periods, and that animal models might prove useful. Lambert, at the recent Lausanne conference (2005), also raised the concern of administering vaccines to adolescent girls, who might develop higher titered antibodies (e.g to PS) than with natural infection by the time they are pregnant any effect on their progeny years after birth would be very hard to detect.

Molecular mimicry between infectious agents and autoimmunity has been reviewed by Wraith, Goldman and Lambert (2003), who have suggested that regulatory factors to common T cell epitopes of protein peptides would most likely be sufficient to prevent possible pathogenic effects. The various aspects of molecular mimicry including vaccines, have also been reviewed in the recent book by Shoenfeld and Rose (2004) and an entire issue in the journal *Autoimmunity* (Fujinami and Cunningham eds. 2006). Some of the pathogenetic mechanisms learned from autoimmunity studies are likely to pertain to those associated to the GENIP hypothesis. Thus, a mother could still produce high levels of harmless antibodies towards her self, but, when these are transplacentally transmitted to the baby, they may still cause damage to the developing neural systems. Of relevance here are cases of Guillain Barré

syndrome noted in infants whose mother had this condition herself during pregnancy (Buchwald et al. 1999; Sladky 2004; Buompadre et al. 2006). The syndrome's occurrence within families (Geleijns et al. 2004) is also of interest.

In view of EVO–DEVO and other considerations, we have focused on PS, primarily sialic acids, as potentially major "pathogenic epitopes". We are concentrating primarily on two types of molecules containing such sialic acid epitopes that occur in various parts of the nervous system, including the brain. Both polysialic acid (PSA) and gangliosides are found in large quantities in early fetal/infant development and in smaller amounts in adults, and both express molecular mimicry with some infectious agents.

Polysialic acids are nine carbon molecules, linked to each other at the $\alpha2-\alpha8$ position in chains of over 50 molecules (Chuong and Edelman 1984; Bruses and Rutishauser 2001). Such PSA molecules are associated with an important nervous system developmental protein—the neural cell adhesion molecule (NCAM), important in a variety of neurological functions in the brain and other parts of the nervous system (Rutishauser 1996; Ni Dhuill et al. 1999). Alterations in such molecules may cause ill-effects during development of the various regions of the nervous system (Cremer et al. 2000). PSA antibodies, or enzymes that degrade PSA, when administered to rodents, will cause several neural dysfunctions (Arami et al. 1996; Rutishauser 1996; Seki and Rutishauser 1998). The NCAM-associated PSA is identical to the PSA in the capsule of two clinically important infectious agents: (i) *E. coli* $K_1$[4]—a major cause of neonatal meningitis and septicemia (Glode et al. 1977; Frosch et al. 1985); (ii) meningococcus B—a major cause of meningitis and septicemia, but primarily in children and adults (Finne et al. 1983). Patients with meningococcus B infections have been found to produce antibodies to NCAM (Nedelec et al. 1990). The molecular mimicry, as demonstrated, for instance, with the cross-reactivity between monoclonal antibodies to the PSA of NCAM, *E. coli* $K_1$ and meningococcus B, has raised concern about including the meningococcus B serogroup with the other four serogroups (Pichichero et al. 2005) in the new protein-conjugated PS meningococcus vaccine. Nevertheless, Robbins' group has proposed recently (Stein et al. 2006) that enough data are available to alleviate such concerns—a position not shared by us, because the data are insufficient, particularly those they review in humans.

The other sialic epitopes are those of gangliosides. Gangliosides are ceramides with 1, 2, 3 or 4 sialic acid epitopes (M, D, T and Q) and variable thin layer chromatography mobility patterns ($_{1,2,3,4}$—$_a$ or $_b$) e.g. $GM_1$ or $GQ_{4b}$ (Rosenberg 1995). Gangliosides are very common, and vary in type and quantity at different stages of brain development in fetal brains of different gestational periods, as well as brains of different ages postnatally (Svennerholm et al. 1989). Gangliosides are also found in other parts of the nervous system, such as peripheral nerves and spinal cord (Svennerholm et al. 1994). They also occur in the placenta, where they may have an immuno-modulatory role (Dyatlovitskaya et al. 1990).

A disease involving peripheral nerves—Guillain-Barré syndrome—has been associated with several infectious agents (Ayala et al. 1999; Hughes et al. 1999; Ang et al. 2000; Nachamkin and Blaser eds. 2000; Press et al. 2001; Butzler 2004). Of these associated agents, *C. jejuni* is the most widely accepted as causal, inducing a neuro-immunopathological disease through the molecular mimicry that exists between its epitopes and those of the nervous system. This very common bacterial diarrheal agent has been found to contain, within its structure, several gangliosides, e.g. GM1. Antibodies to such common sialic acid epitopes can be induced by the bacteria and cause several types of neural dysfunctions [cit. op above]. Furthermore, antibodies to gangliosides have been commonly found in the blood of patients with GBS, although the agent itself cannot be cultured from blood. A genetic predisposition is likely to play an important role, as it is in case of several autoimmune disorders (Shoenfeld and Rose eds. 2004), with several HLA class II alleles being involved with *C. jejuni* (Rees et al. 1995).

*C jejuni* has been associated with about one third of the total number of cases of Guillain Barré syndrome in several studies (Nachamkin and Blaser eds. 2000; Vedeler 2000). It is also associated with an ocular Miller Fisher syndrome with cerebellar involvement, related most commonly to $GQ_{4b}$ antibodies (Rees et al. 1995; Koga et al. 2001). Other agents that have been associated with the Guillain Barré syndrome, albeit studied less well for causality are: *Mycoplasma pneumoniae*, *Hemophilus influenzae*, cytomegalovirus (CMV), Epstein-Barr virus (EBV), herpes simplex virus (HSV) type 1, and varicella virus (as noted earlier, also the swine influenza vaccine of the 1970s, and the more recently used protein-conjugated PS, four serogroup, meningococcus vaccine.)

One explanation for the possible involvement of ganglioside molecular mimicry with viruses has been suggested to be due to "self" ganglioside "pathogenic epitopes" being picked up from host cell membranes during viral replication, and inducing auto-antibodies (Wraith et al. 2003). It is still not possible to rule out that some infectious agents could induce epitope spreading or a general polyactivation, which can be associated more so with certain agents, e.g. Epstein-Barr virus (Poole et al. 2006). It appears therefore helpful to differentiate among different pathogenetic mechanisms by concurrent testing of a variety of different infectious agents, as well as several immune and inflammatory reactants in the same serum.

The oldest and best example of molecular mimicry, associated with a neurological condition—Sydenham's chorea, is that of group A streptococcus (GAS). Recent studies indicate the pathogenesis is related to a ganglioside $GM_1$ (Kirvan et al. 2006). Of interest are findings that the GAS pathogenesis related to rheumatic heart disease, with M-protein molecular mimicry, is likely to be due to T cell autoimmunity, whereas that for the GAS-related NMD—Sydenham's chorea—is related to antibodies to common GAS epitopes reacting mainly with the basal ganglia of the brain (Guilherme et al. 2006; Kirvan et al. 2006). Other possible GAS-related autoimmune conditions, with causality less well ascertained, is the Pediatric Autoimmune Neuropsychiatric disorders (PANDA) associated with GAS, comprising a group of NMD's with different clinical manifestations (Swedo and Grant 2005). Of some common interest is that arthritis, a condition related to GAS, has been associated with Campylobacter (Bremell et al. 1991), and that rheumatoid arthritis has been associated with schizophrenia (Torrey and Yolken 2001).

There is some commonality between the most accepted infectious agents with molecular mimicry to the nervous tissue: *C. jejuni* and GAS. Both contain a ganglioside ($GM_1$), with production of anti-$GM_1$ likely to be responsible for the neural immunopathology. Yet the pathogenesis of the $GM_1$ antibodies appear to differ: (a) in relation to the Guillain Barré syndrome, it appears to be due to complement-mediated antibody cytotoxicity at motor nerve terminals; (b) in relation to Sydenham's chorea the $GM_1$ antibodies appear to affect the physiology of the brain, disturbing primarily cell signaling [cit op above].

It is difficult to explain the differences in the effect of the $GM_1$ antibodies affecting peripheral nerves in one case, and the brain in another. The differences may be due to different effects or the permissiveness of the various blood–nervous system barriers. Several recent books have reviewed important aspects of blood-neural system barriers (Nag 2003; Zheng and Chodobski 2005). It appears that the blood–nervous system barrier is more permissive to blood IgG transfer than are the blood–brain barriers. The blood–CSF barrier is also more permissive to IgG and antibody transfer in fetal life and some time postnatally (~1–2 years). Thus, CSF total protein is known to be higher in the CSF of a premature 6 month gestation baby (up to 200+ mg%), to decrease to around 100 mg% in a 9 month gestation baby, and to reach adult levels of 20–40 mg% around 1–2 years of age. Concurrently, measurements of IgG blood:CSF ratios indicate approximately a similar gradual fall of 100:1 in a 6 month gestational premature infant to adult levels by 1–2 years of age; similar findings have been noted with the passage of IgG specific antibodies. Antibodies of high enough titer would likely be transferred passively for a short distance to the hippocampus or other brain areas bathed by the CSF/ventricular fluid. Several other factors are known to increase the barrier permissiveness of hosts older than infants, including meningitis, encephalitis or head trauma. One wonders whether the trauma of a baby's head passing through the birth canal at delivery may not increase the permissiveness of the blood–brain/CSF barriers, allowing the greater passage of IgG antibodies to the newborn's brain.

Information on the development of cell-to-cell junction blood–brain barriers is difficult to obtain in human fetuses or infants. Active transport of different molecules, including some drugs, occurs by this route (Nag 2003). High levels of bilirubin, which occur with Rh incompatibility, are known to be transferred across this barrier, causing Kernicterus. It should also be appreciated that any ill-effects caused by antibodies to "pathogenic epitopes" would depend at what stage of brain development particular gangliosides or PSA are prevalent, and when particular processes or brain components develop, e.g. neurogenesis develop earlier than synaptogenesis, and the onset of development of the hippocampus, the cerebellum and myelination occurring after mid-gestation (Brody et al. 1987; Kandel et al. 2000; Seress et al. 2001).

The patterning and plasticity of the neonatal cortex is being pursued vigorously in more recent years (Sur and Rubenstein 2005). New EVO–DEVO insights are being obtained related to the evolution and development of the human brain cortex by Christopher Walsh's group (Hill and Walsh 2005; Walsh unpublished results 2006). Certain gene products, found to be lower in association with microcephaly in mice, were also observed to be in lesser amounts in the brain of several lower species than of humans, with the actual amounts correlating with the evolutionary distance between the species tested. Pursuit of such EVO–DEVO discoveries should offer new windows of opportunity to study the human cortex, likely to be relevant to causation of NMD's, as well as to their specific diagnosis, prevention and treatment.

The rationale for testing particular infectious agents, immune and inflammatory factors is partly related to EVO-DEVO and other considerations noted above, as well as results of preliminary studies summarized below. The candidate infectious agents are listed in Figure 2.

### Testability of hypothesis

Testing the GENIP hypothesis requires obtaining serum specimens during pregnancy and at delivery, as well as in newborn infants at birth. The other requirement is to have information on those NMD's, which may take years or even decades to become clinically manifest. Direct and indirect prospective study approaches are available that can provide possible populations of cases and matched controls and the needed specimens to assay putative factors. The small amount of sera available in some cases also necessitates development and application of newer technology. We owe a great debt of gratitude to Danish, Swedish and US collaborating scientists of different disciplines (whose names are listed in the Acknowledgement section). These workers had already identified the populations, some suspect factors, and many of the laboratory test requirements. In turn, our GENIP hypothesis has provided several complementary aspects and concepts: the more likely role of indirect rather than direct infectious neuropathogenesis; the application of an EVO–DEVO perspective, which led to the focus on PS sialic acids as possible "pathogenic epitopes"; a greater list of candidate infectious agents, as well as of other factors, such as antibodies to PSA and Ig classes and subclasses; the benefit of studying MZ and DZ twins with likely different placental transfer of IgG and antibodies; vaccine implications; and other future pursuits of preventive modalities. The preliminary results summarized herein have been obtained mostly by these collaborative scientists. (Interpretation of the results is our own.) Our interactions have helped to place their findings within the context of the more permissive blood–nervous system barriers of the fetus/infant, as well as develop a multifaceted, more comprehensive model that places emphasis on time and space.

### Study populations

There are two "prospective" population study approaches to test for antibodies to putative agents, and correlative pathogenic immune and inflammatory factors:

### *A. Direct type prospective study*

Banked data and sera on pregnant women and babies, with direct follow-up of the progeny to ascertain particularly NMD's have been used to assay for suspect agents and factors. From 1959–1966, the US National Institutes of Health pioneered an approach enrolling women in early pregnancy (primarily from New England and California institutions). In this NIH Perinatal Study, 54,000 live offspring were followed for up to 7 years, primarily to study NMD. Sera had been obtained during pregnancy and at delivery, as well as from cord blood and placentas, and information on obstetric and neonatal complications, twinning, ethnic and other demographic features was obtained. Several reports from the initial 7 year follow-up have been published (e.g. Myrianthopoulos and Chung 1974). More recently, interest has been placed on re-evaluating the behavioral disorders to test for putative agents. With the support of the Stanley Medical Research Institute, schizophrenia cases and appropriate controls, matched for many factors, have been followed in both New England and California, 30 or more years later (the results are discussed below). Plans for enrolling 100,000 more US pregnant women in 2006 to test for the causality of several diseases, with onset in the perinatal period, awaits funding. In the meantime, the Danish Pregnancy Study (Olsen et al. 2001) was initiated with 100,000 Danish pregnant women enrolled from 1998 to 2003, with their offspring being followed for various diseases. Similar specimens, as the earlier NIH study, are available for testing various factors of interest in the blood of mothers and babies. Similar type enrollments with lesser numbers have been, or are planned to be, initiated in other countries.

### *B. More indirect prospective study—linking registries for particular diseases with stored newborn screening blood spots*

Newborn blood spots to assay for a variety of genetic and metabolic disorders have been used for several decades in the US and various countries. Such blood spots have been stored for many years, if not decades, by only a few states, e.g. California in the US, or countries, e.g. Denmark. An issue with blood spots is that they can also be used for DNA characterization, so that there are ethical and other important considerations in regards their uses. For instance, because their use for research was not cleared by the public and proper governmental agencies, testing the blood spots for research projects is not permitted in Sweden.

The approach using the blood spots is to identify individuals in a state or nation who have developed, in later years, particular neuromental or other disorders, often grouped within a registry of the disease. The affected individuals and appropriate matched controls are then linked to their stored blood spots. The Danish State Serum Institute has a bank of over 1 million stored blood spots, in whom they performed, for instance toxoplasma IgM and IgA antibody assays (Sorensen et al. 2002). Focus in recent years in Denmark and California (with CDC) has been placed on schizophrenia, autism and cerebral palsy. We have also recently established a collaboration with Danish and CDC scientists, using a registry of MZ and DZ discordant twins with two NMD's, to study various IgG class and subclasses, as well as several inflammatory factors and antibodies to many of the candidate infectious agents, or

PSA of some of the encapsulated bacteria listed in Figure 2.

### Laboratory assays

Assays have comprised more conventional ones detecting and quantifying antibodies or Ig in serum specimens, such as dye tests for toxoplasma IgG antibodies or ELISA for HSV-2 antibodies. Because of the small amount of blood in the newborn blood spots, a technology that could assay for large numbers of factors was needed. Danish workers used earlier an immunofluorescent technology for blood spots to test for IgA and IgM antibodies to toxoplasma, indicating fetal infection. Over the last few years, the Multiplex Technology has become available (Earley et al. 2002). This allows potentially testing up to 100 analytes (such as antibodies, Ig, inflammatory or neurotropic factors). It has been possible to extract enough sera from one fifth to one eighth of one of the three blood spots, routinely obtained from babies soon after birth. However, rigorous control is needed to ascertain that there is no interference between reactants for each of the analytes being tested. The usual procedure for any Multiplex Assay(s) is to control concordance with more standard serological means, and then test for combinations (Earley et al. 2002). Multiplex applications to test several of the various putative infectious agents or factors (listed in figure 2) have been developed or are under development by collaborators or other workers. They include Multiplex Assays for Ig (Dasso et al. 2002), antibodies to PS antigens (Lal et al. 2004), several cytokines (Kellar and Douglass 2003; Skogstrand et al. 2005), and neurotropic factors associated with autism (Skogstrand et al. 2005; Connolly et al. 2006). Attempts to develop Multiplex assays for antibodies to gangliosides have so far been unsuccessful and the testing of IgG antibodies to eight gangliosides, (in sera obtained by Buka et al. (2001) for schizophrenia and matched controls,) has yielded negative results, using conventional serological tests performed by the collaborating Swedish (Göteborg) laboratory.

### Preliminary results

### *US NIH perinatal study*

The first results with the direct type prospective study approach were published by Buka et al. (2001), who identified 27 New England individuals with schizophrenia and twice the number of matched controls. Maternal sera at delivery were tested by ELISA for antibodies to 6 infectious agents and to the three major Ig classes. Statistically significant differences were found in sera obtained from mothers of babies who developed schizophrenia, as compared to sera from mothers whose progeny had no NMD, in three of the tests: Total IgG and IgM antibodies were higher in the case group, which also had higher titers of antibodies to herpes simplex virus type 2, than in the matched controls (Table I). We tested later the same sera, submitted blindly for testing of IgM antibodies specific to HSV-2, and demonstrated very few positive sera, whether in cases or controls. These results suggest that primary infection, or reactivation of the virus, in the mother did not occur close to the time of delivery. A direct effect of herpes simplex virus type 2 on the brain appeared unlikely from our longtime experience with neonatal herpes simplex virus infection (Nahmias 2004).

New York and California workers, using similar procedures, followed up babies born in the US Perinatal Study, identifying 60 cases of schizophrenia and appropriate controls. These workers tested for influenza virus antibodies, as influenza in pregnant women had been implicated earlier in some, but not all, reports (Brown et al. 2004). The workers found

Table I. Current results obtained in testing banked maternal sera or newborn blood spots–schizophrenia and matched controls.*

| | US-NIH perinatal study (maternal sera at delivery) | | Denmark (blood spots) |
|---|---|---|---|
| | New England | California | |
| Cases vs. controls | ($n = 27$) | ($n = 60$) | ($n > 100$) |
| 1. Herpes simplex virus type 2 antibodies | | | |
| Higher levels of total IgG | $<0.05$ | | |
| Sero-positive for IgG antibodies | NS | | |
| Positive for high levels of IgG antibodies | $<0.05$ | | |
| Positive for IgM antibodies (few) | NS | | |
| 2. Influenza virus antibodies | | | |
| Positive for high levels of influenza virus antibodies | | $<0.05$ | |
| 3. Toxoplasma antibodies | | | |
| sero-positive for IgG antibodies | NS | NS | |
| Positive for high levels of IgG antibodies | NS | $<0.05$ | |
| Positive for IgM antibodies (few) | | NS | $<0.05$ |

*Statistical comparisons are between cases and matched controls without neuro-mental diseases (usually twice the number of cases).

again higher titers of influenza antibodies in cases than controls. It should be noted that influenza, like *C. jejuni*, is rarely found in the blood or brain, and that studies in mice which showed behavioral problems after influenza inoculation, also demonstrated no virus in the brain (Shi et al. 2005). Thus, one explanation for the mouse results is that the antibodies to the virus themselves are responsible for the neural findings.

The results of IgG and IgM antibodies to toxoplasma in the NIH and Danish studies are of particular interest (Table I). In the California cohort, higher titers of toxoplasma IgG antibodies were found in the serum of mothers at delivery, whose pregnancy went on to develop schizophrenia, than in matched controls (Brown et al, 2005). Maternal serum IgM antibodies were not statistically different, suggestive that the maternal infection was not recent. Toxoplasma IgM antibodies were tested in newborn blood spots linked to a large Danish Psychiatric Register, which found significantly more positive tests in cases than controls (Norgaard-Pedersen B et al 2005). IgM antibodies in neonatal blood is strong evidence of a fetal infectious agent that has been able to cross the placental barriers, but does not necessarily prove that the test organism has also crossed the blood-neural system barriers. Neuromental disorders, which are manifest years or decades postnatally, of such congenital infections as toxoplasma, rubella, syphilis or cytomegalovirus, have been generally assumed to be the result of the infectious agent remaining latent in the nervous system. We would suggest that, while this does occur, it may not always be the pathogenetic mechanism. Maternal IgG antibodies (± fetally-produced IgG antibodies to the agent's proteins [Stoll et al 1993]) are also quite likely to be involved.

The importance of the height of antibody levels and not just seropositivity for three infectious agents, has been helpful to validate our model (Figure 3), which accounts for the importance of the blood brain/CSF barriers. Depending on when the levels of transplacentally-acquired antibodies are at their highest, the particular permissiveness in time of the blood–brain/CSF/peripheral nerve barriers, and the coincidence with the development of particular neural processes and components, we conceive of a "window" of optimal risk for pathological effects to occur. Since most of the NMD's occur years or decades after any gestational immunopathological insults, we have postulated also a threshold below which clinical manifest disease will become apparent. For instance, if girls generally are more likely to develop higher thresholds than boys in earlier social and language abilities (Baron-Cohen et al. 2005), they may not be as suspectible to the loss of relevant synapses during programmed pruning at 1-2 years of age. Similarly the synaptic pruning in adolescence may explain the clinical features of schizophrenia becoming first manifest at that age (Feinberg 1982).

### Conclusion

We have presented a hypothesis involving the indirect effects of infectious agents as causes of a proportion of some NMD's. The rationale is based on our prior experience with the more direct effects of infectious agents on the fetus/infant, as well as on evolutionary-developmental (EVO–DEVO) perspectives. Detailed has been the testability of the hypothesis regarding these study populations and laboratory methods suggested by other workers. Based on preliminary results using these methods and other considerations, candidate infectious agents and factors, including certain vaccines, have been identified for further study (Figure 2). A new model portraying the multifaceted factors that must be considered in the hypothesis should assist ongoing and future investigations (Figure 3).

We will end where we began…with the need to prove causality and to develop specific diagnostic tests and preventive measures for, in particular, the more clinically serious and chronic of the NMD's. Otherwise, the millions of babies born every year, who will be affected by one or more NMD's during their lifetime, are destined to experience mild to severe debilitating chronic illnesses for which treatment, even if available in developed countries, would not be a satisfactory global option for the future.

### Acknowledgements

Presented in part at the Conference on Vaccination, Infection and Autoimmunity held in Lausanne, Switzerland, October 26–28, 2005. Dedicated to the memory of Dr Robert A Good, our mentor in the phylogeny and ontogeny of immunology, well before they were linked as EVO–DEVO. Supported in part by the Stanley Medical Research Institute. We are grateful for the discussions and contributions by the many individuals in the US, Denmark and Sweden who helped particularly in providing the population and laboratory study approaches to test our hypothesis and for their past and current collaboration: Niels Bilenberg, Stephen Buka, Pamela Fredman, David Hougaard, Francis Lee, Mads Melbye, Jan Eric Månsson, Bent Nørgaard-Pedersen, Jørn Olsen, Brad Pierce, Abraham Roseberg, Anand Swamy, Fuller Torrey, Paul Thorsen, Robert Vogt and Robert Yolken

### Notes

[1]Although half a decade later, we unfortunately still have little to offer with any of these three types of preventive modalities in the case of congenital cytomegalovirus (CMV) infection.

[2]We have therefore used Gestational Neuro-Immunopathology, or GENIP, to name our hypothesis.

[3]These observations suggest that the terminology used to characterize antibody responses to PS antigens as "T cell-independent" requires revision.

[4]K is German for capsule; $K_1$ was the first one identified possibly because, among 100 or more capsular types of *E. coli* types, it causes about three out of every four cases of *E. coli* newborn meningitis or sepsis.

### References

Ang C, Jacobs B, Laman J, et al. 2000. *Campylobacter jejuni* lipopolysaccharides from Guillain-Barre patients induce IgG anti-GMI antibodies in rabbits. J Neuroimmunol 104:133–138.

Arami S, Jucker M, Schachner M, et al. 1996. The effect of continuous intraventricular infusion of L1 and NCAM antibodies on spatial learning in rats. Behav Brain Res 81:81–87.

Ayala V, Casas C, Ribera J, Cablero J, Oppenheim RW, Esquerda JE. 1999. Specific association of *C. jejuni* like immunoreactivity. J Neurobiol 38:171–190.

Baron-Cohen S, Knickmeyer RC, Belmonte MK. 2005. Sex differences in the brain: Implications for explaining autism. Science 310:819–823.

Benirschke K, Kaufmann P, Baergen RN. 2006. Pathology of the human placenta. 5th ed. New York: Springer Verlag. p 1070.

Bremell T, Bjelle A, Svedhem A. 1991. Rheumatic symptoms following an outbreak of campylobacter enteritis: A five year follow up. Ann Rheum Dis 50:934–938.

Brody BA, Kinney HC, Kloman AS, Gilles FH. 1987. Sequence of central nervous system myelination in human infancy. i. an autopsy study of myelination. J Neuropathol Exp Neurol 46:283–301.

Brown A. 1999. New perspectives on the neurodevelopmental hypothesis of schizophrenia. Psychiatr Ann 29:128–130.

Brown AS, Cohen P, Harkavy-Friedman J, Babulas V, Malaspina D, Gorman JM, Susser ES. 2001. A.E. Bennett research award. Prenatal rubella, premorbid abnormalities, and adult schizophrenia. Biol Psychiatry 49:473–486.

Brown AS, Begg MD, Gravenstein S, Schaefer CA, Wyatt RJ, Bresnahan M, Babulas VP, Susser ES. 2004. Serologic evidence of prenatal influenza in the etiology of schizophrenia. Arch Gen Psychiatry 61:774–780.

Brown AS, Schaefer CA, Quesenberry Jr, CP, Liu L, Babulas VP, Susser ES. 2005. Maternal exposure to toxoplasmosis and risk of schizophrenia in adult offspring. Am J Psychiatry 162:767–773.

Bruses J, Rutishauser U. 2001. Roles, regulation, and mechanism of polysialic acid function during neural development. Biochimie 83:635–643.

Bryan EM. 1977. IgG deficiency in association with placental oedema. Early Hum Dev 1:133–143.

Buchwald B, de Baets M, Luijckx GJ, Toyka KV. 1999. Neonatal Guillain-Barre syndrome: Blocking antibodies transmitted from mother to child. Neurology 53:1246–1253.

Buka SL, Tsuang MT, Torrey EF, Klebanoff MA, Bernstein D, Yolken RH. 2001. Maternal infections and subsequent psychosis among offspring. Arch Gen Psychiatry 58:1032–1037.

Buompadre MC, Ganez LA, Miranda M, Arroyo HA. 2006. Unusual variants of Guillain-Barre syndrome in infancy. Rev Neurol 42:85–90.

Butler JE. 2006. Why I agreed to do this. Dev Comp Immunol 30:1–17.

Butzler JP. 2004. Campylobacter, from obscurity to celebrity. Clin Microbiol Infect 10:868–876.

Carroll S. 2005. Endless forms most beautiful—the new science of EVO–DEVO and the making of the animal kingdom. New York: WW Norton & Co.. p 288.

Castellon SA, Hardy DJ, Hinkin CH, Satz P, Stenquist PK, van Gorp WG, Myers HF, Moore L. 2006. Components of depression in HIV-1 infection: Their differential relationship to neurocognitive performance. J Clin Exp Neuropsychol 28:420–437.

Charney DS, Nestler EJ, editors. 2004. Neurobiology of mental illness. Oxford, NY: Oxford University Press. p 1272.

Chen RT, Glasser JW, Phodes PH, Davis RL, Barlow WE, Thompson RS, Mullooly JP, Black SB, Shinefield HR, Badheim CM, Marcy SM, Ward JI, Wise RP, Wassilak SG, Hadler SC. 1997. Vaccine safety datalink project: A new tool for improving vaccine safety monitoring in the United States. Pediatrics 99:765–773.

Chuong CM, Edelman GM. 1984. Alterations in neural cell adhesion molecules during development of different regions of the nervous system. J Neurosci 4:2354–2368.

Connolly AM, Chez M, Streif EM, Keeling RM, Golumbek PT, Kwon JM, Riviello JJ, Robinson RG, Neuman RJ, Deuel RM. 2006. Brain-derived neurotrophic factor and autoantibodies to neural antigens in sera of children with autistic spectrum disorders, Landau-Kleffner syndrome, and epilepsy. Biol Psychiatry 59:354–363.

Cremer H, Chazal G, Lledo P, et al. 2000. PSA-NCAM: An important regulator of hippocampal plasticity. Int J Dev Neurosci 18:213–220.

Dasso J, Lee J, Bach H, Mage RG. 2002. A comparison of ELISA and flow microsphere-based assays for quantification of immunoglobulins. J Immunol Methods 263:23–33.

Drayna D. 2006. Is our behavior written in our genes? N Engl J Med 354:7–9.

DSM-IV-TR. 2000. Diagnostic and statistical manual of mental disorders. 4th ed. Arlinton, VA: American Psychiatric Publishing Inc..

Dyatlovitskaya EV, Kryukova EV, Suskova VS, Emez VI, Bergelson LD. 1990. Immunomodulatory effects of human placenta gangliosides. Biomed Sci 1:397–400.

Earley MC, Vogt Jr, RF, Shapiro HM, Mandy FF, Kellar KL, Bellisario R, Pass KA, Marti GE, Stewart CC, Hannon WH. 2002. Report from a workshop on multianalyte microsphere assays. Cytometry 50:239–242.

Fagiolo E, Toriani-Terenzi C. 2003. Mechanisms of immunological tolerance loss versus erythrocyte self-antigens and autoimmune hemolytic anemia. Autoimmunity 36:199–204.

Faraone S, Tsuang D, Tsuang M. 1999. Genetics of mental disorder. New York: Guilford Publications Inc.. p 172.

Feinberg I. 1982. Schizophrenia: Due to a fault in programmed synaptic elimination during adolescence? Psychiatr Res 17:319–334.

Finne J, Leinonen M, Makela PH. 1983. Antigenic similarities between brain components and bacteria causing meningitis. Lancet 2:355–357.

Flajnik MF, Du Pasquier L. 2004. Evolution of innate and adaptive immunity: Can we draw a line? Trends Immunol 25:640–644.

Frosch M, Gorgen I, Boulnois GJ, Timmis KN, Bitter-Suermann D. 1985. NZB mouse system for production of monoclonal antibodies to weak bacterial antigens: Isolation of an IgG antibody to the polysaccharide capsules of *Escherichia coli* K1 and group B meningococci. Proc Natl Acad Sci USA 82:1194–1198.

Fujinami R, Cunningham M, editors. 2006. Autoimmunity. 39., p 1–77.

Geleijns K, Brouwer BA, Jacobs BC, Houwing-Duistermaat JJ, van Duijn CM, van Doorn PA. 2004. The occurrence of Guillain-Barre syndrome within families. Neurology 63:1747–1750.

Glode MP, Sutton A, Robbins JB, McCracken GH, Gotschlich EC, Kaijser B, Hanson LA. 1977. Neonatal meningitis due of *Escherichia coli* K1. J Infect Dis 136:S93–S97.

Goodman FR. 2003. Congenital abnormalities of body patterning: embryology revisited. Lancet 362:651–662.

Guilherme L, Kalil J, Cunningham M. 2006. Molecular mimicry in the autoimmune pathogenesis of rheumatic heart disease. Autoimmunity 39:31–39.

Harrison PJ, Law AJ. 2006. Neuregulin 1 and schizophrenia: Genetics, gene expression, and neurobiology. Biol Psychiatry, Jan 24; [Epub ahead of print].

Hill RS, Walsh CA. 2005. Molecular insights into human brain evolution. Nature 437:64–67.

Hughes R, Hadden R, Gregson N, et al. 1999. Pathogenesis of Guillain-Barré syndrome. J Neuroimmunol 100:74–97.

Insel BJ, Brown AS, Bresnahan MA, Shaefer CA, Susser ES. 2005. Maternal-fetal blood incompability and the risk of schizophrenia in offspring. 80:331–342.

Iwahashi K, Watanabe M, Nakamura K, Suwaki H, Nakaya T, Nakamura Y, Takahashi H, Ikuta K. 1997. Clinical investigation of the relationship between Borna disease virus (BDV) infection and schizophrenia in 67 patients in Japan. Acta Psychiatr Scand 96:412–415.

Kandel E, Schwartz J, Jessell T, editors. 2000. Principles of neural science. 4th ed., New York: McGraw Hill. p 1195.

Kandel EM. 2006. In search of memory The emergence of a new science of mind. New York: WW Norton & Company. p 510.

Karlsson H, Bachmann S, Schroder J, McArthur J, Torrey EF, Yolken RH. 2001. Retroviral RNA identified in the cerebrospinal fluids and brains of individuals with schizophrenia. Proc Natl Acad Sci USA 98:4634–4639.

Kasahara M, Suzuki T, Pasquier LD. 2004. On the origins of the adaptive immune system: Novel insights from invertebrates and cold-blooded vertebrates. Trends Immunol 25(2):105–111.

Kellar KL, Douglass JP. 2003. Multiplexed microsphere-based flow cytometric immunoassays for human cytokines. J Immunol Methods 279:277–285.

Kirvan CA, Swedo SE, Kurahara D, Cunningham MW. 2006. Streptococcal mimicry and antibody-mediated cell signaling in the pathogenesis of Sydenham's chorea. Autoimmunity 39:21–29.

Klein JO, Helms CM. 2005. Strengthening the supply of routinely administered vaccines in the United States: Progress and problems—2005. Clin Infect Dis 42(3):S145–S150.

Kobrynski L, Sousa AO, Nahmias AJ, Lee FK. 2005. Cutting edge: Antibody production to pneumococcal polysaccharides requires CD1 molecules and CD8 + T cells. J Immunol 174: 1787–1790.

Koga M, Yuki N, Tai T, Hirata K. 2001. Miller-Fisher syndrome and haemophilus influenzae infection. Neurology 57:686–691.

Lal G, Balmer P, Joseph H, Dawson M, Borrow R. 2004. Development and evaluation of tetraplex flow cytometric assay for quantitation of serum antibodies to Neisseria meningitidis serogroups A, C, Y and W-135. Clin Diagn Lab Immunol 11: 272-9.

Law AJ, Lipska BK, Weickert CS, Hyde TM, Straub RE, Hashimoto R, Harrison PJ, Kleinman JE, Weinberger DR. 2006. Neuregulin 1 transcripts are differentially expressed in schizophrenia and regulated by 5′ SNPs associated with the disease. Proc Natl Acad Sci USA, Apr 17; [Epub ahead of print].

Lee FK, Nahmias AJ, Nahmias DG, McDougall JS. 1983. Demonstration of virus particles within immune complexes by electron microscopy. J Virol Methods 7:167–181.

Lee FK, Nahmias AJ, Lowery SA, Nesheim S, Reef S, Thompson SE, Oleske J, Vahlne A, Czerkinsky C. 1989. ELISPOT—A new approach to studying the dynamics of virus-immune system interaction for diagnosis and monitoring of HIV infection. AIDS Res Hum Retroviruses 5:517–523.

Lee FK, Nahmias AJ, Spira T, Keyserling H, Lowery S, Reimer C, Black C, Stoll B, Czerkinsky C. 1991. Enumeration of human peripheral blood lymphocytes secreting immunoglobulins of major classes and subclasses in healthy children and adults. J Clin Immunol 11:213–218.

Lynch VJ, Wagner GP. 2006. The birth of the uterus. Nat Hist 114:36–41.

Malek A, Sager R, Kuhn P, Nicolaides KH, Schneider H. 1996. Evolution of maternofetal transport of immunoglobulins during human pregnancy. Am J Reprod Immunol 36:248–255.

Marchalonis JJ, Adelman MK, Schluter SF, Ramsland PA. 2006. The antibody repertoire in evolution: Chance, selection, and continuity. Dev Comp Immunol 30:223–247.

Menez-Bautista R, Fikrig SM, Pahwa S, Sarangadharan MG, Stoneburner RL. 1986. Monozygotic twins discordant for the acquired immunodeficiency syndrome. Am J Dis Child 140:678–679.

MMWR., Guillain-Barré Syndrome Among Recipients of Menactra® Meningococcal Conjugate Vaccine—United States, June-July 2005. MMWR Dispatch 54:1023–1025. Available from: http://www.cdc.gov/mmwr

MMWR. Update: Guillan-Barré Syndrome Among Recipients of Menactra® Meningococcal Conjugate Vaccine—Unted States, October 2005–Febrary 2006. MMWR Dispatch 55: 364-365. Available from: http://www.cdc.go/mmwr

Molina V, Shoenfeld Y. 2005. Infection, vaccines and other environmental triggers of autoimmunity. Autoimmunity 38:235–245.

Musher DM. 2006. Pneumococcal vaccine-direct and indirect ("herd") effects. N Engl J Med 354:1522–1524.

Myrianthopoulos NC, Chung CS. 1974. Congenital malformations in singletons: Epidemiological survey. Birth Defects: Orig Artic Ser March of Dimes X(11):1–58.

Nachamkin I, Blaser MJ, editors. 2000. Campylobacter. 2nd ed. Washington, DC: ASM Press.

Nag S. 2003. The blood–brain barrier, biology and research protocols. New Jersey: Humana Press. p 572.

Nahmias A, Alford C, Korones S. 1970a. Infection of the newborn with herpes simplex viruses. In: Schulman I, editor. Advances in pediatrics., p 185–226.

Nahmias AJ, Josey WE, Naib ZM. 1970b. Neonatal herpetic infection—comparison with other congenital infections Proceedings of 5th International Congress of Infectious Diseases. Vienna, Austria:, p 355–362.

Nahmias A, Walls K, Stewart J, Flynn W, Herrmann K. 1971. The TORCH complex—perinatal infections associated with toxoplasma and rubella, cytomegalo- and herpes simplex viruses. Pediatr Res 5:405–406.

Nahmias A, Reanney D. 1977. The evolution of viruses. Annu Rev Ecol Syst 8:29–49.

Nahmias A, Visintine A. 1979. Role of infectious agents in birth defects: An overview of still unresolved problems. In: Mulunsky A, editor. Genetic disorders of the fetus. New York: Plenum Press. p 569–589.

Nahmias A, O'Reilly R, editors. 1981. Immunology of human infection Part I: Bacteria, mycoplasmae, chlamydiae, and fungi. New York: Plenum Press. p 651.

Nahmias A, O'Reilly R, editors. 1982. Immunology of human infection Part II: Viruses and parasites. New York: Plenum Press. p 603.

Nahmias A, Keyserling H. 1984. Neonatal herpes simplex in context of the TORCH complex. In: Holmes K, Weisner P, Sparling F, March P, editors. Sexually transmitted diseases. New York: McGraw Hill. p 816–826.

Nahmias AJ, Lee FK, Beckman Nahmias S. 1990. Sero-epidemiological and -sociological patterns of herpes simplex virus infection in the world. Scand J Inf Dis S69:19–36.

Nahmias AJ, Stoll BJ, Hale E, Ibegbu CC, Keyserling H, Innis-Whitehouse W, Holmes R, Spira T, Czerkinsky C, Lee FK. 1991. IgA-secreting cells in the blood of premature and term infants: Normal development and effect of intrauterine infections. Adv Exp Med Biol 310:59–69.

Nahmias A, et al. 1992. Immunologie des infections virales aux differents niveaux d'immunitié du foetus et du nouveau né. In: Lejeune C, editor. Virus et Grossesse. Dauville. p 105–136.

Nahmias A, Panigel M, Schwartz D. 1994a. The eight most frequent blood-borne infectious agents affecting the placenta and fetus—a synoptic review. Trophoblast Res 8:193–213.

Nahmias AJ, Ibegbu C, Lee FK, Spira T. 1994b. The development of the immune system. Clin Immunol Immunopathol 71:2–7.

Nahmias A, Kourtis A. 1997. The great balancing acts: The placenta, the mother, the fetus and infectious agents. In: Stoll BJ, Weisman LE, editors. Clinical perinatology. Philadelphia: W.B. Saunders. p 497–521.

Nahmias A, Abramowsky C, Dobronyi I, et al. 1998a. Infection and Immunity at the Maternal-Placental-Fetal Interface. Trophoblast Res 12:102–124.

Nahmias A, Lee F, Ibegbu C, et al. 1998b. Immunology of HIV infection in the fetus and newborn. In: Shearer W, Moffenson L, editors. Immunology & Allergy clinics of north america. 18. 2, p 401–419.

Nahmias AJ. 2004. Neonatal HSV infection part i. continuing challenges. Herpes 11:33–37.

Nature 2002. Neuroscience. Nature 5:1019–1097.

Nedelec J, Boucraut J, Garnier J, et al. 1990. Evidence for autoimmune antibodies directed against embryonic neural cell adhesion molecules (N-CAM) in patients with group B meningitis. J Neuroimmunol 29:49–56.

Ni Dhuill C, Fox G, Pittock S, O'Connell A, Murphy K, Regan C. 1999. Polysialylated neural cell adhesion molecule expression in the dentate gyrus of the human hippocampal formation from infancy to old age. J Neurosci Res 55:99–106.

Nørgaard-Pedersen B, et al. Unpublished results 2005.

Olness K. 2003. Effects on brain development leading to cognitive impairment: A worldwide epidemic. J Dev Behav Pediatr 24:120–130.

Olsen J, Melbye M, Olsen SF, Sorensen TI, Aaby P, Andersen AM, Taxbol D, Hansen KD, Juhl M, Schow TB, Sorensen HT, Andresen J, Mortensen EL, Olesen AW, Sondergaard C. 2001. The danish national birth cohort–its background, structure and aim. Scand J Public Health 29:300–307.

Palca J. 1991. HIV risk higher for first-born twins. Science 254:1729.

Pichichero M, Casey J, Blatter M, Rothstein E, Ryall R, Bybel M, Gilmet G, Papa T. 2005. Comparative trial of the safety and immunogenicity of quadrivalent (A, C, Y, W-135) meningococcal polysaccharide-diphtheria conjugate vaccine versus quadrivalent polysaccharide vaccine in two- to ten-year-old children. Pediatr Infect Dis J 24:57–62.

Poole BD, Scofield RH, Harley JB, James JA. 2006. Epstein-Barr virus and molecular mimicry in systemic lupus erythematosus. Autoimmunity 39:63–70.

Press R, Mata S, Lolli F, Zhu J, Andersson T, Link H. 2001. Temporal profile of anti-ganglioside antibodies and their relation to clinical parameters and treatment in Guillain-Barre syndrome. J Neurol Sci 190:41–47.

Rees J, Vaughn R, Kondeatis E, et al. 1995. HLA class II alleles in Guillain-Barré and Miller-Fisher syndrome. J Neuroimmunol 62:53.

Remington J, Klein J, et al. 2006. Infectious diseases of the fetus and newborn infant. 6th ed. Philadelphia: W.B. Saunders Co.. p 1328.

Rosenberg A. 1995. Biology of sialic acids. New York: Plenum Press. p 394.

Rutishauser U. 1996. Polysialic acid and the regulation of cell interactions. Curr Opin Cell Biol 8:679–684.

Schinazi R, Nahmias A, editors. 1988. AIDS in children, adolescents and heterosexual adults New York: Elsevier Science Publisher. p 212.

Seki T, Rutishauser U. 1998. Removal of polysialic acid-neural cell adhesion molecule induces aberrant mossy fiber innervation and ectopic synaptogenesis in the hippocampus. J Neurosci 18:3757–3766.

Seress L, Abraham H, Tornoczky T, Kosztolanyi G. 2001. Cell formation in the human hippocampal formation from mid-gestation to the late postnatal period. Neuroscience 105:831–843.

Shi L, Tu N, Patterson PH. 2005. Maternal influenza infection is likely to alter fetal brain development indirectly: The virus is not detected in the fetus. Int J Dev Neurosci 23:299–305.

Shoenfeld Y, Rose N, editors. 2004. Infection and autoimmunity. New York: Elsevier. p 768.

Skogstrand K, Thorsen P, Norgaard-Pedersen B, Schendel DE, Sorensen LC, Hougaard DM. 2005. Simultaneous measurement of 25 inflammatory markers and neurotrophins in neonatal dried blood spots by immunoassay with xmap technology. Clin Chem 51:1854–1866.

Sladky JT. 2004. Guillain-Barre syndrome in children. J Child Neurol 19:191–200.

Snapper CM, Shen Y, Khan AQ, Colino J, Zelazowski P, Mond JJ, Gause WC, Wu ZQ. 2001. Distinct types of T-cell help for the induction of a humoral immune response to Streptococcus pneumoniae. Trends Immunol 22:308–311.

Sorensen T, Spenter J, Jaliashvili I, Christiansen M, Norgaard-Pedersen B, Petersen E. 2002. Automated time-resolved immunofluorometric assay for toxoplasma gondii-specific IgM and IgA antibodies: Study of more than 130,000 filter-paper blood-spot samples from newborns. Clin Chem 48:1981–1986.

Stein DM, Robbins J, Miller MA, Lin FY, Schneerson R. 2006. Are antibodies to the capsular polysaccharide of Neisseria meningitidis group B and *Escherichia coli* K1 associated with immunopathology? Vaccine 24:221–228.

Stoll BJ, Lee FK, Hale E, Schwartz D, Holmes R, Ashby R, Czerkinsky C, Nahmias AJ. 1993. Immunoglobulin secretion by the normal and infected newborn infant. J Pediatr 122:780–786.

Stoll BJ. 1997. The global impact of neonatal infection. Clin Perinatol 24:1–21.

Sur M, Rubenstein JL. 2005. Patterning and plasticity of the cerebral cortex. Science 310:805–810.

Svennerholm L, Bostrom K, Fredman P, Mansson JE, Rosengren B, Rynmark BM. 1989. Human brain gangliosides: Developmental changes from early fetal stage to advanced age. Biochim Biophys Acta 1005:109–117.

Svennerholm L, Bostrom K, Fredman P, Jungbjer B, Lekman A, Mansson JE, Rynmark BM. 1994. Gangliosides and allied glycosphingolipids in human peripheral nerve and spinal cord. Biochim Biophys Acta 1214:115–123.

Swedo SE, Grant PJ. 2005. Annotation: PANDAS: A model for human autoimmune disease. J Child Psychol Psychiatry 46:227–234.

The World Health Report. Mental Health: New Understanding, New Hope, Available from: World Health Report Archives http/www.who.int/whr

Torrey FE, Yolken RH. 2000. Familial and genetic mechanisms in schizophrenia. Brain Res Brain Res Rev 31:113–117.

Torrey EF, Yolken RH. 2001. The schizophrenia-rheumatoid arthritis connection: Infectious, immune, or both? Brain Behav Immun 15:401–410.

Vedeler CA. 2000. Inflammatory neuropathies: Update. Curr Opin Neurol 13:305–309.

Walsh CA, Unpublished results

Weinberger DR. 1996. On the plausibility of "the neurodevelopmental hypothesis" of schizophrenia. Neuropsychopharmacology 14:1S–11S.

Wraith DC, Goldman M, Lambert PH. 2003. Vaccination and autoimmune disease: What is the evidence? Lancet 362:1659–1666.

Wurtz R. 1998. Psychiatric diseases presenting as infectious diseases. Clin Infect Dis 26:924–932.

Zheng W, Chodobski A. 2005. The blood–cerebrospinal fluid barrier. Boca Raton, FL: Taylor & Francis.

